# The effectiveness of an oral opioid rescue medication algorithm for postoperative pain management compared to PCIA: A cohort analysis

**DOI:** 10.1007/s00101-020-00806-6

**Published:** 2020-07-02

**Authors:** J. Erlenwein, M. I. Emons, F. Petzke, M. Quintel, I. Staboulidou, M. Przemeck

**Affiliations:** 1 Department of Anesthesiology, University Hospital, Georg August University of Göttingen, Robert-Koch-Str. 40, 37075 Göttingen, Germany; 2 Fetal Medicine Center Hannover, Podbielskistraße 122, 30177 Hannover, Germany; 3 Department of Anesthesiology and Intensive Care, Annastift, Hannover, Anna-von-Borries-Straße 1–7, 30625 Hannover, Germany

**Keywords:** Patient controlled analgesia, Acute pain service, Standard operating procedures, Long-acting opioids, Patientenkontrollierte Analgesie, Akutschmerzdienst, Behandlungsstandards, Retardierte Opioide

## Abstract

**Background:**

Standard protocols or algorithms are considered essential to ensure adequate analgesia. Germany has widely adopted postoperative protocols for pain management including oral opioids for rescue medication, but the effectiveness of such protocols has only been evaluated longitudinally in a before and after setting. The aim of this cohort analysis was to compare the effectiveness of an oral opioid rescue medication algorithm for postoperative management of pain to the gold standard of patient-controlled intravenous analgesia (PCIA).

**Material and methods:**

This study compared cohorts of patients of two prospective observational studies undergoing elective total hip replacement. After surgery patients received piritramide to achieve a pain score of ≤3 on the numeric rating scale (NRS 0–10). A protocol was started consisting of oral long-acting oxycodone and ibuprofen (basic analgesia). Cohort 1 (C1, 126 patients) additionally received an oral opioid rescue medication (hydromorphone) when reporting pain >3 on the NRS. Cohort 2 (C2, 88 patients) was provided with an opioid by PCIA (piritramide) for opioid rescue medication. Primary endpoints were pain intensity at rest, during movement, and maximum pain intensity within the first 24 h postoperative. Secondary endpoints were opioid consumption, functional outcome and patient satisfaction with pain management.

**Results:**

Pain during movement and maximum pain intensity were higher in C1 compared to C2: pain on movement median 1st–3rd quartile: 6 (3.75–8) vs. 5 (3–7), *p* = 0.023; maximum pain intensity: 7 (5–9) vs. 5 (3–8), *p* = 0.008. There were no differences in pain intensity at rest or between women and men in either group. The mean opioid consumption in all patients (combined PACU, baseline, and rescue medication; mean ± SD mg ME) was 126.6 ± 51.8 mg oral ME (median 120 (87.47–154.25) mg ME). Total opioid consumption was lower in C1 than C2 (117 ± 46 mg vs 140 ± 56 mg, *p* = 0.002) due to differences in rescue opioids (C1: 57 ± 37 mg ME, C2: 73 ± 43 mg ME, *p* = 0.006, Z = −2.730). Basic analgesia opioid use was comparable (C1: 54 ± 31 mg ME, C2: 60 ± 36 mg ME, *p* = 0.288, Z = −1.063). There were no differences in respect to the addition of non-opioids and reported quality of mobilization, sleep, frequency of nausea and vomiting, or general satisfaction with pain management.

**Conclusion:**

In this study PCIA provided a better reduction of pain intensity, when compared to a standardized protocol with oral opioid rescue medication. This effect was associated with increased opioid consumption. There were no differences in frequencies of opioid side effects. This study was a retrospective analysis of two cohorts of a major project. As with all retrospective studies, our analysis has several limitations to consider. Data can only represent the observation of clinical practice. It cannot reflect the quality of a statement of a randomized controlled trial. Observational studies do not permit conclusions on causal relationships.

## Abstract (de)

**Hintergrund:**

Standardprotokolle oder Algorithmen sind für eine adäquate Schmerztherapie essenziell. Protokolle zur postoperativen Schmerztherapie mit oralen Opioiden zur Bedarfsanalgesie sind in deutschen Krankenhäusern sehr weit verbreitet und etabliert, die Wirksamkeit solcher Protokolle wurde bisher jedoch nur längsschnittlich in einer Vorher-Nachher-Situation bewertet. Ziel dieser Kohorten-Analyse war es, die Wirksamkeit eines Bedarfsalgorithmus mit oralen unretardierten Opioiden zur postoperativen Schmerzbehandlung mit dem Goldstandard der patientenkontrollierten intravenösen Analgesie („patient controlled intravenous analgesia“ [PCIA]) zu vergleichen.

**Material und Methoden:**

In dieser Studie wurden die Kohorten von zwei prospektiven Beobachtungsstudien verglichen, bei denen ein elektiver Hüftgelenkersatz durchgeführt worden war: Postoperativ wurden die Patienten zunächst mit Piritramid titriert, um eine Schmerzintensität von ≤3 auf der numerischen Rating-Skala (NRS, 0–10) zu erreichen. Anschließend wurde ein Protokoll bestehend aus retardiertem oralem Oxycodon und Ibuprofen gestartet (Basisanalgesie). Kohorte 1 (C1, 126 Patienten) erhielt zusätzlich bei Bedarf (NRS >3) ein unretardiertes orales Opioid (Hydromorphon); Kohorte 2 (C2, 88 Patienten) erhielt stattdessen eine Opioid-PCIA (Piritramid). Primäre Endpunkte waren die Schmerzintensität in Ruhe, während der Bewegung und die maximale Schmerzintensität innerhalb der ersten 24 h postoperativ. Sekundäre Endpunkte waren der Opioidkonsum, funktionelle Ergebnisse und die Zufriedenheit der Patienten mit der Schmerzbehandlung.

**Ergebnisse:**

Der Schmerz bei Bewegung und die maximale Schmerzintensität waren in Gruppe C1 höher verglichen mit C2 (Median 1.–3. Quartil: 6 [3,75–8] vs. 5 [3–7], *p* = 0,023; maximale Schmerzintensität: 7 [5–9] vs. 5 [3–8], *p* = 0,008). Es gab keine Unterschiede bei der Schmerzintensität in Ruhe, hinsichtlich Mobilisation und Schlafqualität, Häufigkeit von Übelkeit und Erbrechen sowie allgemeiner Zufriedenheit mit der Schmerztherapie.

**Schlussfolgerung:**

In Bezug auf die Reduzierung der Schmerzintensität war die PCIA einem standardisierten Protokoll mit oraler Opioid-Bedarfsmedikation überlegen. Dieser Effekt war mit einem erhöhten Opioidkonsum verbunden. Es gab keinen Unterschied in der Häufigkeit von Opioid-Nebenwirkungen. Diese retrospektive Studie enthält deutliche Limitationen, wie sie viele retrospektive klinische Analysen aufweisen. Sie spiegelt nicht die Qualität einer Aussage einer randomisierten kontrollierten Studie wider. Beobachtungsstudien erlauben keine Schlussfolgerungen oder kausalen Zusammenhänge.

## Background

Caregivers have focused on improvement in postoperative pain management for several years; however, for some areas quality of pain management remains inadequate [1, 22]. Previous surveys found that patients undergoing procedures without the use of advanced analgesia concepts, such as peripheral pain catheters, or epidural analgesia, are more likely to suffer from insufficient pain relief [13, 22]. Since patients with peripheral pain catheters or neuraxial analgesia are commonly monitored by an acute pain service (APS) and patients without are not, this could be a potential explanation for the observed differences. For practical and economic reasons, the involvement of an APS for all patients seems challenging and might not be necessary [19, 20, 36]. Novel approaches and concepts to supplement an APS are therefore of great interest and potential impact [10, 20, 34]. Standard protocols or algorithms, which enable regular nursing floor staff to react to the patients’ needs, including an adequate response to an acute increase in pain, are considered essential to ensure sufficient analgesia [10, 27]. Despite significant heterogeneity, these concepts typically include instructions for a rescue medication to be administered in cases of insufficient pain relief [2, 5, 6, 14, 15, 18, 27, 32, 35]. Germany has widely adopted protocols including oral opioids for rescue medication [6]. Ease of administration and independence from any sort of catheter or device, thought to impair the patients’ mobilization or mobility, are obvious advantages of this approach. Despite the widespread adoption of protocols including oral opioids as rescue medication in most German hospitals, the effectiveness of such protocols has only been evaluated longitudinally in a before and after setting, meaning before and after their implementation within a clinical setting [2, 3, 5, 6, 9, 14, 15, 18, 27, 32, 35]. Patient-controlled intravenous analgesia (PCIA) has been the technique of choice for comparisons for years and is still considered the gold standard [21].

## Objective

The aim of this analysis was to compare the effectiveness of a standardized algorithm to control the process of application of rescue medication based on oral short-acting opioids to the administration of short-acting opioids via patient-controlled intravenous analgesia (PCIA).

## Material and methods

### Study cohorts

In this analysis of two cohorts of previously conducted prospective, observational trials we compared the effectiveness of a standardized algorithm based on an oral short-acting opioid postoperative pain management in comparison with PCIA. Both arms primarily aimed to investigate the influence of different pain-related characteristics in patients with osteoarthritis of the hip scheduled for total hip replacement on postoperative pain intensity, analgesic consumption and functional outcome [8, 16]. Protocols were approved by the Ethics Committee of the University Hospital of Göttingen, Germany, and written informed consent was obtained from participants. Studies were conducted according to the recommendations of the Declaration of Helsinki.

Data were collected consecutively from July to October 2012 (cohort 1, C1) and from April to July 2013 (cohort 2, C2) at a single center (Orthopedic University Hospital Annastift, Hannover). The established clinical standards of the hospital were executed for postoperative pain management [10] and were not changed for the strictly observational studies; however, between the two studies one detail of the hospitals standard postoperative clinical analgesic regimen was changed. Patients in cohort 1 received an oral opioid pain rescue medication, patients in cohort 2 received PCIA for the first 24 postoperative hours.

Primary endpoints were pain intensity at rest, pain intensity during movement and maximum pain intensity within the first 24 postoperative hours. Secondary endpoints were opioid consumption in morphine equivalents within the first 24 h, functional outcomes (quality of mobilization and sleep, incidence of tiredness/sedation, nausea and vomiting), and patient satisfaction with pain management. All patients were admitted to the hospital and consented to participate in the study on the day before surgery. In both arms, we collected data from hospital admission to the first postoperative day.

### Inclusion/exclusion criteria

Patients were included consecutively if they were at least 18 years old, able to understand the study information and the questionnaires and provided written informed consent. Furthermore, the indications for total hip replacement had to be primary osteoarthritis of the hip. No stratification for age or gender was applied. In order to minimize variations in pain intensity in the early postoperative phase due to different anesthetic techniques, all participants receiving spinal or regional anesthesia were excluded. Furthermore, we excluded patients diagnosed with dementia or any other major neurologic condition, active drug abuse, or preoperative opioid consumption of >30 mg of morphine equivalents. We did not include patients participating in other studies simultaneously. To ensure more homogeneous groups, patients with acute hip pain due to femoral head necrosis were not included. The C2 cohort underwent electroencephalogram (EEG) recording on the day prior to surgery. Therefore, patients with a significant neurologic disease, such as stroke or epilepsy were excluded in C2.

### Clinical procedure

On the evening prior to surgery and the following morning all patients received 20–30 mg dipotassium chlorazepate. General anesthesia was performed according to clinical standards and induced by a remifentanil (1–1.5 µg/kg for 3 min) and propofol bolus (1–2 mg/kg). Orotracheal intubation was facilitated by 0.5 mg/kg atracurium. Anesthesia was maintained with sevoflurane 0.7–1.0 MAC or propofol 3.5–4.5 mg/kg and h, along with remifentanil (0.15–0.25 µg/kg and min). According to hospital standard, depth of anesthesia was monitored by bispectral index (BIS®, Covidien, Medtronic, Minneapolis, MN, USA). During the final stages of the operation, i.e. implantation of the femoral shaft, 0.1 mg/kg piritramide and 15 mg/kg metamizole (if contraindicated, an equivalent amount of paracetamol) were administered intravenously. All patients were extubated and transferred to the postanesthesia care unit (PACU).

### Postoperative pain management

**Fig. 1:**
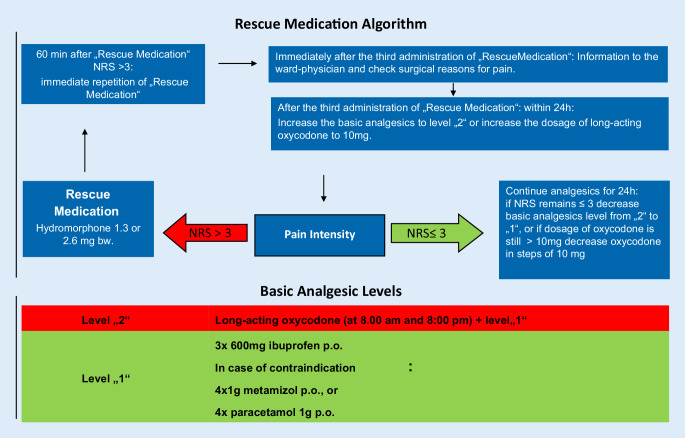
Standard oral opioid algorithm in the Orthopedic University Hospital Annastift, Hannover, Germany

Upon PACU arrival, pain was treated with repeated doses of 3.75 mg piritramide IV to reach a pain score of ≤3 on the numeric rating scale (NRS 0–10) in all patients. While still in the PACU, all patients (C1 and C2) received 10 or 20 mg long-acting oxycodone orally (age-adapted and/or weight-adapted: 10 mg if weight <60 kg and/or age >70 years, 20 mg for all others) and 600 mg ibuprofen orally for basic analgesia. Once on the regular nursing ward, patients received oral oxycodone twice daily and ibuprofen 600 mg three times a day, following the standardized protocol (Fig. 1). In the case of contraindications for ibuprofen, patients received 4 × 1 g metamizole/dipyrone, or 4 × 1 g paracetamol [10]. The initial amount of long-acting oxycodone administered on the regular nursing ward was the same as given in the PACU. Pain intensity (NRS) was assessed three times a day in routine and every time the patient reported pain.

The C1 patients were offered 1.3–2.6 mg hydromorphone orally (age and weight-adjusted) when NRS was >3. Pain intensity was reassessed 60 min after administration of an analgesic. If pain intensity remained >3 on the NRS, a second hydromorphone dose was offered. If pain persisted after further 60 min, a third dose of hydromorphone was administered, and the physician was consulted. Oxycodone dose was increased by 10 mg twice daily when 3 doses of hydromorphone were given within 24 h. Whenever the patients’ pain intensity scored <3 over the past 24 h, each dose of long-acting oxycodone was reduced by 10 mg (Fig. 1).

Patients in the “C2” group received PCIA with piritramide (Perfusor fm PCA, B. Braun, Melsungen, Germany; single dose 2 mg, lockout 10 min, limit 30 mg/4h) for 24 h instead of the oral hydromorphone. The PCIA was initiated in the PACU as soon as patients were able to operate the system. All patients were instructed about their respective postoperative analgesia protocol including pain assessment using NRS. “C1” patients were informed of the availability of supplemental doses of analgesics if required, while “C2” patients were instructed in the use of the PCIA system.

### Pain history

The patients’ preoperative pain history was assessed by a standardized protocol based on the German pain questionnaire (localization, duration, pain intensity on an 11-point NRS, and prehospital analgesic consumption) [26].

### Assessment of postoperative pain and functional limitations

Postoperative pain was assessed 24 h after surgery by the quality improvement in postoperative pain therapy (QUIPS) questionnaire, a validated German outcome assessment tool, including questions about pain intensity during movement, maximum pain intensity and pain intensity at rest over the previous 24 h (NRS: 0 = no pain to 10 = worst pain) [24, 25]. In addition, questions about patient satisfaction with their postoperative pain management (NRS: 0 = not satisfied to 15 = very satisfied), the presence of postoperative functional limitations, and further need for analgesics were included (mobilization, coughing/deep breathing, night sleep and side effects—nausea, vomiting and tiredness: yes/no).

### Analgesic consumption

Due to interindividual variability the preoperative opioid consumption between the two cohorts was compared in two steps: 1) opioid intake “yes”/“no” and 2) “no”/“only as required”/“regular daily dosage <30 mg morphine equivalent”).

To compare the postoperative analgesic consumption, opioid doses were expressed as oral morphine equivalent (ME; conversion factor to morphine: piritramide 1.5, hydromorphone 0.13, oxycodone 0.75; intravenous vs. oral morphine 3:1). For the current analysis, the postoperative opioid consumption was calculated for the time from surgery including the first postoperative day, which includes opioids received in the PACU.

### Statistical analysis

Percentages were rounded to the nearest whole number. Categorical variables are presented either as percentages in groups or as median with first and third quartiles. Continuous attributes are described using the mean and standard deviation. Correlations between pain scores (cumulative value for intensity during movement, maximum pain intensity and pain intensity at rest), opioid consumption and patient satisfaction were calculated with Kendall-Tau‑b. Group comparisons with categorical and continuous characteristics were analyzed using non-parametric tests (Mann–Whitney *U*-test). The distributions of frequencies of dichotomous attributes in groups were described using the Pearson χ^2^-test.

A *p*-value of 0.05 was considered statistically significant. Results were not adjusted for multiple testing for single hypothesis.

## Results

### Inclusions and exclusions

Overall, 172 patients were scheduled for total hip replacement in the first period and 175 were scheduled in the second period.

#### Cohort 1 (C1).

Of the 172 patients screened, 11 declined participation, 10 met exclusion criteria (3 were enrolled in other studies, 1 was <18 years old, 1 spoke no German, 3 were hospitalized out of the clinical routine on the day of surgery, 1 had active drug abuse, and 1 had dementia). A further 8 were excluded due to planned spinal anesthesia and 3 patients due to preoperative opioid consumption with daily doses >30 mg ME. After inclusion and recording of baseline parameters 18 patients had to be excluded because the surgery was cancelled, postoperative delirium occurred, or they had acute (<6 months) hip pain due to femoral head necrosis.

#### Cohort 2 (C2).

Of the 175 patients screened, 24 patients declined to participate; 1 was less than 18 years old; 1 was excluded because he was already enrolled in another study; and 1 was excluded because both hips were replaced in one session. One patient could not be included because he was a prisoner and informed consent could not be obtained. 13 patients were excluded due to planned spinal anesthesia, 4 patients due to preoperative opioid use with daily doses >30 mg ME, and 1 due to drug abuse. A further 17 patients were not included due to relevant neurologic conditions in their medical history. Another 18 patients were excluded after signing the informed consent because the surgery was cancelled or postponed. One patient was excluded because a postoperative transitional syndrome occurred, and 5 patients did not finish the PCIA therapy according to the study protocol for 24 h and/or pain intensity was not assessed after 24 h.

### Patient preoperative characteristics

A total of 214 patients were included in this analysis (C1: *n* = 126; C2 *n* = 88). Even though the two groups did not differ with respect to age and body mass index (BMI), the proportion of men was higher in C1 than in C2. Due to this difference in distribution, the results for postoperative pain intensity and analgesic consumption were also presented separately for gender.

There were no differences in the duration of hip pain before surgery between the two groups of patients (Table 1). Furthermore, there were no differences between the groups with respect to preoperative hip pain intensity in the last 3 months before admission to the hospital and pain intensity on the day of admission. No differences were present with respect to preoperative analgesic consumption (Table 1).

**Table 1:** Patient characteristics

	Total	C1	C2	Statistical analysis
	*n* = 214	*n* = 126	*n* = 88	
Women (%)	52	41	58	*p* = 0.013, χ^2^ = 6.345
Men (%)	48	59	42	
Age (years)	64.0 ± 12.3	63.3 ± 12.4	65.1 ± 12	n.s., Z = −1.015
Body mass index (BMI)	28.0 ± 5.1	28.3 ± 5.3	27.7 ± 4.8	n.s., Z = −0.990
*Duration of hip pain (%)*				
1–6 months	12	8	15	n.s., Z = −0.775
6–12 months	15	15	16	
1–2 years	21	21	21	
2–5 years	29	32	24	
>5 years	23	22	24	
*Preoperative hip pain intensity (NRS)*				
Mean hip pain intensity in the 3 months before admission	6 (5–7)	5 (5–7)	6 (5–7)	n.s., Z = −0.751
Hip pain intensity on the day of admission	5 (3–7)	5 (2–7)	6 (3–8)	n.s., Z = −0.186
*Preoperative opioid consumption (%)*				
“Yes”	16	18	14	n.s., χ^2^ = 0.567
*Preoperative opioid dosage (%)*				
None	84	83	86	n.s., Z = −0.696
“As required”	5	6	4	
Daily dosage <30 mg morphine equivalent	11	11	10	

Numerical variables are presented as mean ± SD. Variables on the numeric rating scale (NRS) are presented as median and 1st and 3rd quartile, categorical variables are presented in %

### Postoperative pain intensity and opioid consumption

**Fig. 2:**
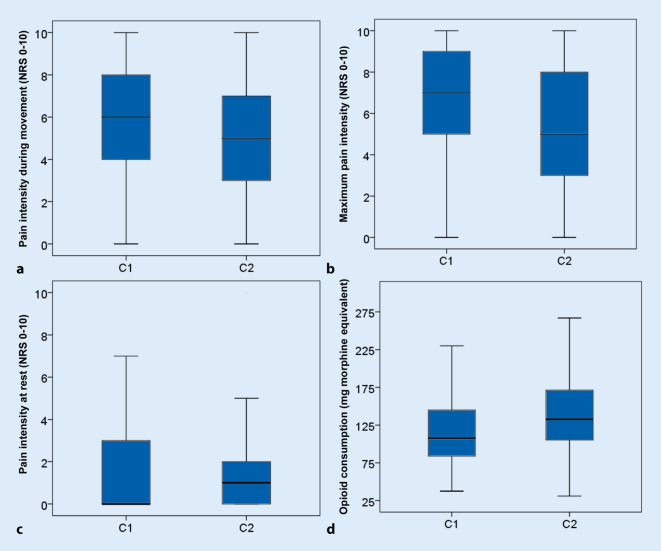
Postoperative pain intensity and opioid consumption: **a** pain intensity during movement (*p* = 0.023, Z = −2.266), **b** maximum pain intensity (*p* = 0.008, Z = −2.666), **c** pain intensity at rest (n.s. Z = −0.114.), **d** opioid consumption (*p* = 0.001, Z = −3.876)

**Fig. 3:**
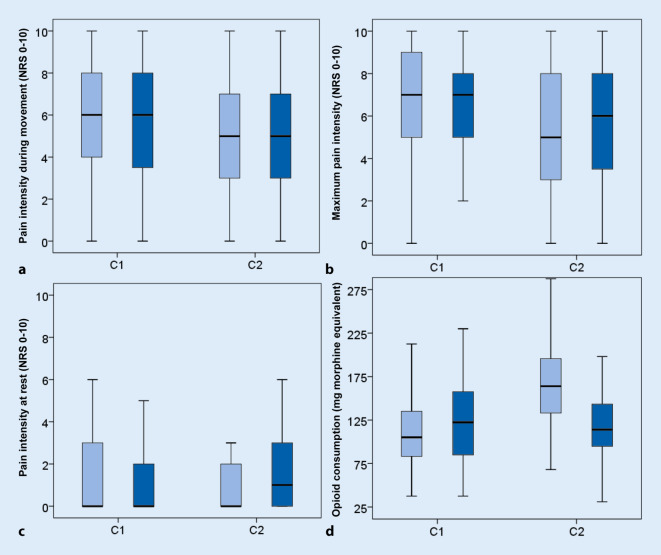
Postoperative pain intensity and opioid consumption separated by sex (*light blue bar* = male, *dark blue bar* = female): **a** pain intensity during movement (C1 n.s., Z = −0.535, C2 n.s., Z = −0.204), **b** maximum pain intensity (C1 n.s., Z = −0.293; C2 n.s., Z = −0.221), **c** pain intensity at rest (C1 n.s., Z = −0.459; C2 n.s., Z = −1.129), **d** opioid consumption (C1 n.s., Z = −0.960; C2 *p* < 0.001, Z = −3.833)

On the day following surgery, patients with PCIA (C2) reported lower maximum pain intensity and lower pain during movement. There were no differences in pain intensity at rest (Fig. 2). No differences in pain intensity were found between women and men in either group (Fig. 3).

The mean opioid consumption in all patients (combined PACU, long-acting opioid medication, and rescue medication/PCIA, C1 and C2) was 126.6 ± 51.8 mg oral ME (median 120 [87.47–154.25] mg ME) over the first 24 h. Overall opioid consumption was higher in patients with PCIA (C1: 117 ± 46 mg ME, C2: 140 ± 56 mg ME, Fig. 2). The difference was attributable to a more frequent use of rescue opioids (C1: 57 ± 37 mg ME, C2: 73 ± 43 mg ME, *p* = 0.006, Z = −2.730). There was no significant difference in the amount of long-acting opioids (C1: 54 ± 31 mg ME; C2: 60 ± 36 mg ME, *p* = 0.288, Z = −1.063).

Men with PCIA had a significantly higher overall opioid consumption than women (C2: 166.7 ± 58.1 vs. 120.9 ± 47.3 mg) while there was no difference between women and men treated with an oral opioid rescue medication algorithm (Fig. 3).

Within the cohort of patients with PCIA (C2) total amount of opioids delivered via PCIA was not statistically different between women and men (32.0 ± 27.8 mg ME vs. 36.6 ± 27.1 mg ME, n.s., Z = −0.944).

There were no significant differences between the cohorts regarding the use of additional non-opioids, 79% in C1 and 81% in C2 received an antiphlogistic non-opioid (ibuprofen, χ^2^ = 0.656). If contraindicated most patients received 4 × 1 g metamizole/dipyrone; only 8 patients alternatively received 4 × 1 g paracetamol.

### Pain-related functional outcome and side effects

Despite the differences in pain intensity, both cohorts demonstrated comparable outcomes of pain-related functional restrictions. There were no differences in quality of sleep or the patients’ mood. Although a high proportion of patients suffered nausea or vomiting, there were no differences between patients treated with oral opioid rescue medication algorithm or PCIA (Table 2).

**Table 2:** Pain-related functional outcome and side effects

	Total	C1	C2	Statistical analysis	C1 women/men	Statistical analysis	C2 women/men	Statistical analysis
	*n* = 214	*n* = 126	*n* = 88		*n* = 51/75		*n* = 51/37	
Is pain interfering with your mobility or movement? (“yes”)	83	83	83	n.s., χ^2^ = 0.006	81/84	n.s., χ^2^ = 0.665	12/24	n. s., Χ^2^ = 2.392
Are you experiencing pain when you cough or breathe deeply? (“yes”)	10	8	13	n.s., χ^2^ = 1.219	6/9	n.s., χ^2^ = 0.495	12/14	n. s., χ^2^ = 0.060
Were you woken up by pain last night? (“yes”)	38	42	32	n.s., χ^2^ = 2.312	41/43	n.s., χ^2^ = 0.028	29/35	n. s., χ^2^ = 0.324
Is pain interfering with your mood? (“yes”)	22	25	17	n.s., χ^2^ = 2.108	14/33	*p* = 0.013, χ^2^ = 6.160	12/24	n. s., χ^2^ = 2.392
Would you have liked to have received more pain medication? (“yes”)	10	13	7	n.s., χ^2^ = 1,942	12/13	n.s., χ^2^ = 0.067	6/8	*n*.s, χ^2^ = 0.167
Have you felt very tired since surgery? (“yes”)	84	85	82	n.s., χ^2^ = 0.365	77/91	*p* = 0.041, χ^2^ = 4.778	86/76	n. s., χ^2^ = 1.619
Have you felt nauseous since surgery? (“yes”)	43	45	40	n.s., χ^2^ = 0.631	35/52	n.s., χ^2^ = 3.420	45/32	n. s., χ^2^ = 1.436
Have you vomited since surgery? (“yes”)	25	25	26	n.s., χ^2^ = 0.065	20/28	n.s., χ^2^ = 1.153	31/19	n. s., χ^2^ = 1.723

All variables are presented in %

There were no functional differences between women and men treated with PCIA. The gender-specific subanalysis of the item “mood” within the cohorts revealed that in “C1” more men than women felt that pain interfered with their mood, whereas more women than men felt tired (Table 2).

### Patient satisfaction

Patient reported satisfaction with pain management was high in both cohorts and not different (NRS 0–15; C1: 13 (10.75–15), C2: 13 (12–14), n.s., Z = −1.279). We found no gender-specific distinctions in overall satisfaction (C1 men/women: ns, Z = −1.379, C2 men/women: ns, Z = −0.343). Variation in pain intensity and opioid consumption did also not affect the patients’ wishes to receive more pain medication (Table 2).

### Correlations

Not surprisingly, pain intensity was correlated with rescue opioid consumption (both oral and PCIA) after surgery (Kendall-Tau-b = 0.224, *p* = 0.001), as well as weakly correlated with overall opioid consumption within 48 h (Kendall-Tau-b = 0.111, *p* = 0.018). We also found a mild negative correlation between pain intensity and patients’ satisfaction (Kendall-Tau-b = −0.320, *p* > 0.001). There was no significant correlation between the pain intensities and the amount of long-acting opioid medication.

These results were similar in the cohort-specific analysis, except for an absence of correlation between pain intensity and opioid consumption in the PCIA cohort (C1 cumulative pain intensity and 48h opioid consumption: Kendall-Tau-b = 0.166, *p* = 0.008, C1 cumulative pain intensity and opioid rescue medication: Kendall-Tau-b = 0.246, *p* = 0.001, C2 cumulative pain intensity and opioid rescue medication: Kendall-Tau-b = 0.257, *p* = 0.01, cumulative pain intensity and satisfaction: Kendall-Tau-b = −0.334, *p* > 0.001; C2 cumulative pain intensity and opioid-consumption: ns, cumulative pain intensity and satisfaction: Kendall-Tau-b = −0.316, *p* > 0.001).

## Discussion

We explored the effectiveness of an oral opioid rescue medication algorithm compared to PCIA in two consecutive cohorts of patients undergoing total hip replacement. Patients receiving PCIA reported lower maximum pain intensity and lower pain during movement. Lower pain intensities were associated with higher overall opioid consumption in patients with PCIA compared to patients who received oral opioid rescue medications; however, there were no differences in pain at rest, functional limitations and overall satisfaction. Therefore, despite the smaller reduction in pain intensity, an oral opioid rescue medication algorithm might still lead to satisfactory results in terms of postoperative mobilization and patient satisfaction.

### Benefits of pain management with PCIA

Our results suggest that the advantage of PCIA treatment, specifically lower pain intensity in the first postoperative hours after total hip replacement, is achieved by higher opioid consumption. This finding is in line with previous studies, summarized in a recent Cochrane review [23]. In this review patients with PCIA had better outcomes regarding pain intensity, but also required more opioids than the control group [23].

In contrast, there was no correlation between cumulative pain intensity and opioid consumption in patients with the PCIA. This might highlight the importance of individual aspects, such as age, problems with intravenous access, and technical competence, leading to insufficient pain management with PCIA in some patients [30]. Thus, the personalization of analgesic medication to the individual patient and needs (especially pain intensity) should be considered an essential element of any postoperative pain concept.

For standardized protocols with short-acting oral opioids for rescue medication, triggers or minimum thresholds for pain intensity in combination with clear instructions and a predefined timeline should ensure a pathway of action with respect to dynamic needs, and compensate for the reduced patient autonomy and increased barriers compared to PCIA.

Despite greater pain relief with PCIA, we found no differences in functional outcomes (quality of mobilization and sleep) or side effects (incidence of nausea and vomiting). This is supported by other studies reaching similar conclusions [23]. In these studies, pruritus was the only side effect that occurred more frequently in patients with PCIA.

### Standardization of postoperative pain management

Implementation of standardized protocols has improved pain management for various surgical procedures and clinical settings as shown in studies using preintervention/postintervention measurements [2, 5, 6, 14, 15, 18, 27, 32, 35]. Standardized protocols seem to be pivotal elements in the management of acute and postoperative pain, especially when a dedicated APS is not readily available [13, 22]; however, these investigations were not based on a single intervention or randomized controlled trials, but reflect a whole range of procedural and structural changes [2, 5, 6, 14, 15, 18, 27, 32, 35]. Thus, a multifactorial mix of effects likely contributes to the improvements demonstrated in these studies. In addition, standardized protocols for postoperative pain management vary in terms of actual content and objectives [6, 7]. Therefore, the ward staff’s capacity to actively administer rescue analgesia triggered by patient reports of pain may differ between these standards.

A recent analysis of the factual contents of standardized postoperative pain protocols showed that many of these concepts did not even include provision of a rescue medication [6]. Some concepts regulated the process of pain management in the sense of an overall clinical pathway; others regulated only the administration of a specified medication to a particular group of patients, without providing a pathway of action in the case of insufficient effects and/or increasing pain intensity [7]. The protocol used in the current study, met five minimum requirements for postoperative pain management for protocols and algorithms, suggested based on a recent analysis of protocols for postoperative pain management [6]:

1. Immediate availability of a potent, fast-acting substance on demand (typically a short-acting opioid)
2. Control of effectiveness after a predefined period following administration of rescue medication
3. Possibility of repeating this rescue medication after a predefined period
4. Basic analgesia, including a non-opioid and an opioid (ideally long acting)
5. Further instructions if pain relief remains insufficient, despite rescue medication

### Gender-specific comparison

Gender differences in pain perception and tolerance have been a focus of research for a considerable time. [28]. While some studies detected gender specific differences in the response to pharmacological and non-pharmacological pain management, others did not [11].

Analyzing men and women separately in our study revealed that women treated with PCIA received and/or required fewer opioids than men. This finding may be explained by different psychological characteristics, or pharmacokinetic differences. Also, men without PCIA were less likely to actively demand an oral analgesic in case of relevant pain compared to women [29]; however, there were no significant differences in pain intensity between women and men receiving the same analgesic rescue medication regimen (oral opioid rescue medication algorithm or PCIA), challenging the overall clinical relevance of this difference in opioid use.

### Strengths

Data for both cohorts were collected on patients undergoing a surgical procedure with a high grade of standardization in a single recruiting hospital. All patients received the same baseline analgesics. The only difference between the cohorts was the method of providing rescue medication. Since the group with PCIA used this in addition to the same baseline medication, valid insights about the effectiveness of the oral rescue medication algorithm could be gained. Another strength of our study is the assessment of functional outcomes in addition to pain intensity and analgesic consumption. Assessment of functional outcomes in pain management should be a standard in both trials for postoperative pain management and monitoring of clinical practice. Outcome measures like QUIPS and/or Pain OUT (improvement in postoperative PAIN OUTcome) offer this possibility [24, 38].

### Limitations

This retrospective study was a prespecified subgroup analysis of two prospective observational trials. Data collection was not blinded. The current results thus have relevant potential for bias or lack of control for confounding variables. As with all non-randomized trials, our results should not be interpreted as a causal relationship, but rather an association. The size of the two cohorts clearly differed, which could potentially affect the results. The differences in cohort size are a result of limited time windows for consecutive and prospective patient recruiting. Since this study was a subgroup analysis of previously recorded data, the number of patients included was determined by the time period of recruitment, and no additional effect or sample size was performed. In addition, the applicability and validity of traditional *p*-values has been questioned recently for retrospective observational studies [4, 33]; however, to our knowledge there is currently no consensus for alternative analytic procedures [12, 17].

Furthermore, the study arms were not primarily designed and conducted to assess the effectiveness of an analgesic protocol. On the other hand, there were no other conceptual differences in the management of these patients than the difference in rescue analgesia (oral vs. PCIA). Throughout the study, multiple surgeons cared for patients in both cohorts. Thus, from a clinical viewpoint, the analysis reflects and supports the feasibility of a standardized protocol with oral opioid rescue medication.

A further limitation is the assessment of opioid consumption. Only the total amount was assessed in the C group, without separation into basic analgesia (with long-acting opioids) and rescue administration (with short-acting opioids). Thus, no differentiated conclusions on patient behavior in the first cohort are possible. Furthermore, the pharmacokinetics of intravenous and oral opioids is substantially different, which might have influenced the effectiveness of the opioid medication irrespective of the dosing and the opioid used. Several publications have questioned the validity of conversions of opioid equivalents by highlighting relevant interindividual variations; however, this is a widely accepted approach to compare opioid regimens, which were highly standardized in this study despite the use of different opioids [31, 37]. In addition, an overall effect over 24 h was measured, so that the specific pharmacokinetics probably had little influence on the assessment, apart from maximum pain intensity.

While the standard clinical analgesia algorithm at the study-hospital combined different opioids for intravenous therapy, as well as oral rescue medication and basic analgesia, advantages for limiting the number of active substances used (ideally to one opioid available in several application forms) have been described.

## Conclusion

The use of PCIA as rescue medication is superior to a standardized concept with an oral opioid rescue medication algorithm for reducing pain intensity; however, greater pain relief in patients with PCIA goes along with increased opioid consumption. In terms of functional aspects and pain intensity at rest, we found no differences. This clinical study was a retrospective analysis of previously collected prospective data. It therefore has inherent limitations and potential bias. There was no prospective calculation of effect or sample size, the two study arms were not primarily designed and conducted to assess the effectiveness of analgesic protocols, differences in cohort size occurred due to limited time windows of consecutive recruiting, and a control for confounding variables is lacking. Even though data were collected on patients undergoing one specific surgical procedure with a high grade of standardization in a single recruiting hospital and with the same overall management in both cohorts (with the exception of rescue analgesia with oral opioids versus PCIA), study results only represent a clinical observation and not the quality and validity of a randomized controlled trial. Observational studies do not permit conclusions on causal relationships.

## Abbreviations

APS: Acute pain service
BIS: Bispectral index
BMI: Body mass index
EEG: Electroencephalogram
IV: Intravenous
ME: Morphine equivalent
NRS: Numeric rating scale
PACU: Postanesthesia care unit
Pain OUT: Improvement in postoperative pain outcome
PCIA: Patient-controlled intravenous analgesia
QUIPS: Quality improvement in postoperative pain management
